# Pure T-cell mediated rejection following kidney transplant according to response to treatment

**DOI:** 10.1371/journal.pone.0256898

**Published:** 2021-09-03

**Authors:** Hyunwook Kwon, Young Hoon Kim, Youngmin Ko, Seong Jun Lim, Joo Hee Jung, Chung Hee Baek, Hyosang Kim, Su-Kil Park, Sung Shin, Yong-Pil Cho

**Affiliations:** 1 Division of Kidney and Pancreas Transplantation, Department of Surgery, Asan Medical Center, University of Ulsan College of Medicine, Seoul, Korea; 2 Division of Nephrology, Department of Internal Medicine, Asan Medical Center, University of Ulsan College of Medicine, Seoul, Korea; 3 Division of Vascular Surgery, Department of Surgery, Asan Medical Center, University of Ulsan College of Medicine, Seoul, Korea; Istituto Di Ricerche Farmacologiche Mario Negri, ITALY

## Abstract

The focus of studies on kidney transplantation (KT) has largely shifted from T-cell mediated rejection (TCMR) to antibody-mediated rejection (ABMR). However, there are still cases of pure acute TCMR in histological reports, even after a long time following transplant. We thus evaluated the impact of pure TCMR on graft survival (GS) according to treatment response. We also performed molecular diagnosis using a molecular microscope diagnostic system on a separate group of 23 patients. A total of 63 patients were divided into non-responders (N = 22) and responders (N = 44). Non-response to rejection treatment was significantly associated with the following factors: glomerular filtration rate (GFR) at biopsy, ΔGFR, TCMR within one year, t score, and IF/TA score. We also found that non-responder vs. responder (OR = 3.31; *P =* 0.036) and lower GFR at biopsy (OR = 0.56; *P =* 0.026) were independent risk factors of graft failure. The responders had a significantly superior overall GS rate compared with the non-responders (*P* = 0.004). Molecular assessment showed a good correlation with histologic diagnosis in ABMR, but not in TCMR. Solitary TCMR was a significant risk factor of graft failure in patients who did not respond to rejection treatment.

## Introduction

The first Banff classification for diagnosis of allograft rejection in kidney transplantation (KT), which was first introduced in 1991, mainly stipulated T-cell mediated rejection (TCMR) rather than antibody-mediated rejection (ABMR), and characterized TCMR by T-cell infiltration in the graft interstitial tissues and tubules [1]. Over the last two decades, however, the impact of TCMR on graft survival (GS) has gradually decreased due to the advances in immunosuppressive agents [2]. Pure TCMR activity commonly occurs during the early periods after transplant and are rare after five years after transplant [2]. In addition, the treatment of acute TCMR using corticosteroids, the currently recommended treatment of choice, shows good responses in most cases [3]. Moreover, a prospective study reported that pure TCMR was no longer the cause of graft failure [4]. On the contrary, the number of studies highlighting the importance of ABMR and donor-specific antibody (DSA) in long-term GS has explosively increased [4–6]. Particularly, one study reported that ABMR was the primary cause of graft failure and accounted for 64% of the included patients with pathological evidence [4]. Collectively speaking, the focus in KT research has largely shifted from TCMR to ABMR.

In clinical practice, however, clinicians still encounter cases of pure acute TCMR without evidence of ABMR in histological reports, even after a long time following transplant. Recent studies on acute TCMR were mainly focused on the impact of TCMR combined with ABMR and chronic active TCMR [7–9], and studies on acute pure TCMR, especially those occurring after more than one year after transplant, have been rarely performed [10]. Thus, even as studies are shifting toward ABMR, there is an unmet need to evaluate the clinical implication of pure TCMR according to the time of rejection and response to treatment.

To evaluate the impact of pure TCMR on clinical outcomes, the histologic diagnosis of pure TCMR should be first ensured. However, the diagnosis of TCMR by Banff classification using interstitial inflammation and tubulitis relies on arbitrary thresholds and nonspecific lesions [11]. Moreover, interobserver error and poor reproducibility in histologic TCMR diagnoses have been issued as well [12]. In this aspect, molecular tests have emerged to reduce the ambiguity in histologic diagnosis of TCMR [13].

In this study, we evaluated the impact of pure TCMR on GS according to treatment response. We also performed molecular diagnosis using a molecular microscope diagnostic system (MMDx) biopsy assessment in an additional group analysis to determine the accuracy of the histologic diagnosis of pure TCMR [13].

## Materials and methods

### Patients

This was a retrospective, single-center study carried out at Asan Medical Center in Seoul, Republic of Korea. Among 276 patients diagnosed with acute rejection at our center between 2009 and 2017, we included 63 patients with first episode of pure acute TCMR. Patients with evidence of ABMR (N = 142), borderline TCMR (N = 65), and non-compliance (N = 6) were excluded. A total of 63 patients were thus included and divided into non-responders and responders according to response to rejection treatment. AMC IRB waived informed consent due to the retrospective nature of this study using data from patients’ medical records. The molecular diagnosis and histologic diagnosis were conducted simultaneously on a separate group of 23 patients who agreed to participate in our study with written informed consent between April 2018 and September 2018. The clinical and research activities being reported are consistent with the Principles of the Declaration of Istanbul, as outlined in the Declaration of Istanbul on Organ Trafficking and Transplant Tourism. None of the transplant donors were from vulnerable populations, and all donors or next of kin freely provided written informed consent. Organs/tissues were procured only at registered institutions with The Korean Network for Organ Sharing [14]. The Asan Medical Center institutional review board (IRB organizations’ IORG number: IORG0009892 / Federal wide assurance number: 00005513) approved this study (AMC IRB number: 2018–0369).

### Diagnosis and treatment of rejection treatment

Biopsies were only performed when there was a suspicion of acute rejection and were reviewed by experienced pathologists at our center following the most up-to-date Banff criteria at the time of diagnosis [15]. Each biopsy sample was reviewed by at least two pathologists, but not necessarily independently. All biopsies were reviewed with clinicians prior to final reporting to share clinical information with pathologists. C4d staining was carried out in all specimens. HLA typing for detecting HLA-A, -B, -C, -DRB1, and -DQB1 was carried out by sequence-based typing. The DSA was measured at the time of biopsy by using HLA class I and II single antigen bead (LABScreen, One Lambda, Canoga Park, CA, USA) and detected by a Luminex system (LabScan100, One Lambda). At our center, methylprednisolone (mPD) pulse therapy was used as the primary treatment for acute TCMR; the total doses of mPD were 1500 mg for grade I TCMR and 2500 mg for grade II–III TCMR, and were gradually tapered to 16 mg per day until the second week and reduced to 4–8 mg per day thereafter. Anti-thymocyte globulin (ATG) (thymoglobulin, Genzyme, Cambridge, MA, USA) was used in patients with side effects against mPD.

### Molecular diagnosis: Microarray assessment

In addition to the four core specimens obtained for histological diagnosis, we obtained one additional biopsy core from 23 patients for molecular diagnosis. The extra biopsy samples for molecular diagnoses were immediately placed in RNAlater (Qiagen, Mississauga, Canada) to stabilize cellular RNA and sent to the Alberta Transplant Applied Genomics Centre (ATAGC, University of Alberta) for microarray analysis using MMDx [16] The molecular diagnosis provided classifier scores with positive cut-off values of ≥ 0.1 for TCMR and ≥ 0.2 for ABMR. TCMR MMDx scores were classified as normal, mild, moderate, and severe using the cut-off values of 0.1, 0.3, and 0.6. ABMR MMDx scores were classified as normal, mild, and severe using the cut-off values of 0.2 and 0.5 [16]. Finally, we compared the results of histological results conducted at our center with the MMDx reports.

### Definition

Responders were defined as recipients whose serum glomerular filtration rate (GFR) decreased by less than 30% from baseline value at three months after rejection treatment, which was estimated using the modification of diet in renal disease equation [10, 17]. The baseline eGFR was determined by averaging the last three results of GFR (average standard deviation of three GFR values = 3.62) before decreasing due to rejection. The GFR at biopsy and three months after treatment were the results of the given time point. The cut-off value of 30% was used because this was the GFR that began to show significant differences in long-term GS after adjusting risk factors associated with GS. Accordingly, patients whose post-treatment GFR value decreased by more than 30% from the pre-treatment value were categorized as non-responders. The primary outcome was GS, which was defined as the time from the first episode of TCMR to return to dialysis or pre-emptive re-transplantation. Secondary outcomes were the cumulative incidence of recurrent rejection and correlation between histologic and molecular diagnosis. We excluded patients with non-adherence to immunosuppressants according to the medical records. The clinicians reported non-compliance based on reports from patients or family members. Clinicians asked patients whether immunosuppressants were taken properly if, during follow-up, drug concentrations were abnormally low, if patients did not regularly turn up to outpatient clinic appointments, and if there were discrepancies between pill counts and prescriptions.

### Statistical analysis

Categorical variables were evaluated by the chi-squared test or Fisher’s exact test as appropriate and presented as counts and percentages. Normally distributed continuous variables were analyzed by the Student’s *t-*test and presented as mean ± standard deviation, and non-normally distributed continuous variables were compared with Mann–Whitney *U* test and presented as medians and interquartile ranges (IQRs). Analysis of variance (ANOVA) and Kruskal–Wallis test were used to analyze variables involving three groups, as appropriate. The GS and incidence of recurrent rejection rate after treatment were evaluated with the Kaplan–Meier method and compared using the log-rank test. The risk factors for non-response were analyzed using univariate and multivariate logistic regression. The factors associated with graft failure were evaluated by Cox proportional hazards regression analysis with forward selection. Bootstrap analysis was performed to determine if there were any overfitting variables. All statistical analyses were conducted in SPSS Statistics for Windows, version 18.0 (SPSS Inc., Chicago, IL, USA) and *P-*value of 0.05 was considered as the cut-off for statistical significance.

## Results and discussion

### Patient demographic

Of the 63 patients included in this study, 22 (35%) and 41 (65%) patients were categorized as non-responders and responders, respectively. The baseline demographics and clinical characteristics of the patients are shown in **Table 1.** The non-responders had a significantly higher median baseline GFR (83.8 mL/min/1.73 m^2^, IQR 65.8–93.3) than the responders (60.3 mL/min/1.73 m^2^, IQR 51.3–66.7; *P =* 0.027). The proportion of patients who developed TCMR within one year after transplantation was significantly higher in the responder group (56%) than in the non-responder group (18%; *P =* 0.004). Otherwise, there were no significant differences between the two groups.

**Table 1:** Baseline and clinical characteristics of the study patients.

	Non-responders (N = 22, 35%)	Responders* (N = 41, 65%)	*P*-value
Age (years)	48.8 ± 12.3	45.0 ± 12.4	0.24
Female sex	5 (22.7)	10 (24.4)	0.99
Diabetes mellitus	9 (40.9)	9 (22.0)	0.15
Body mass index (kg/m^2^)	23.6 ± 3.8	23.0 ± 3.5	0.49
Donor age (years)	43.0 ± 14.5	43.9 ± 8.8	0.76
Cold ischemic time (minutes)	45.2 ± 29.7	52.3 ± 32.0	0.39
Cadaveric donor	2 (9.1)	7 (17.1)	0.39
Retransplantation	0 (0.0)	1 (2.4)	1.00
ABO incompatible	2 (9.1)	6 (14.6)	0.70
HLA-A, B, DR mismatch	3.3 ± 1.5	3.5 ± 1.8	0.56
0% ≤ PRA class I < 20%	22 (100.0)	39 (95.1)	0.99
20% ≤ PRA class I ≤ 100%	0 (0.0)	2 (4.8)	
0% ≤ PRA class II < 20%	22 (100.0)	38 (92.7)	0.70
20% ≤ PRA class II ≤ 100%	0 (0.0)	3 (7.3)	
Pre-transplant DSA positive	0 (0.0)	1 (2.4)	0.46
Induction			0.40
Basiliximab	22 (100.0)	38 (92.7)	
Thymoglobulin	0 (0.0)	3 (7.3)	
Calcineurin inhibitor			0.44
acrolimus	10 (45.5)	23 (56.1)	
Cyclosporin	12 (54.5)	18 (43.9)	
Duration of dialysis (months)	26.3 ± 27.0	26.1 ± 48.3	0.98
Baseline GFR (mL/min/1.73 m^2^)	83.8 (65.8–93.3)	60.3 (51.3–66.7)	0.027
Rejection within 1 year after transplantation	4 (18.2)	23 (56.1)	0.004

### Clinical and histological characteristics at the time of TCMR

**Fig 1:**
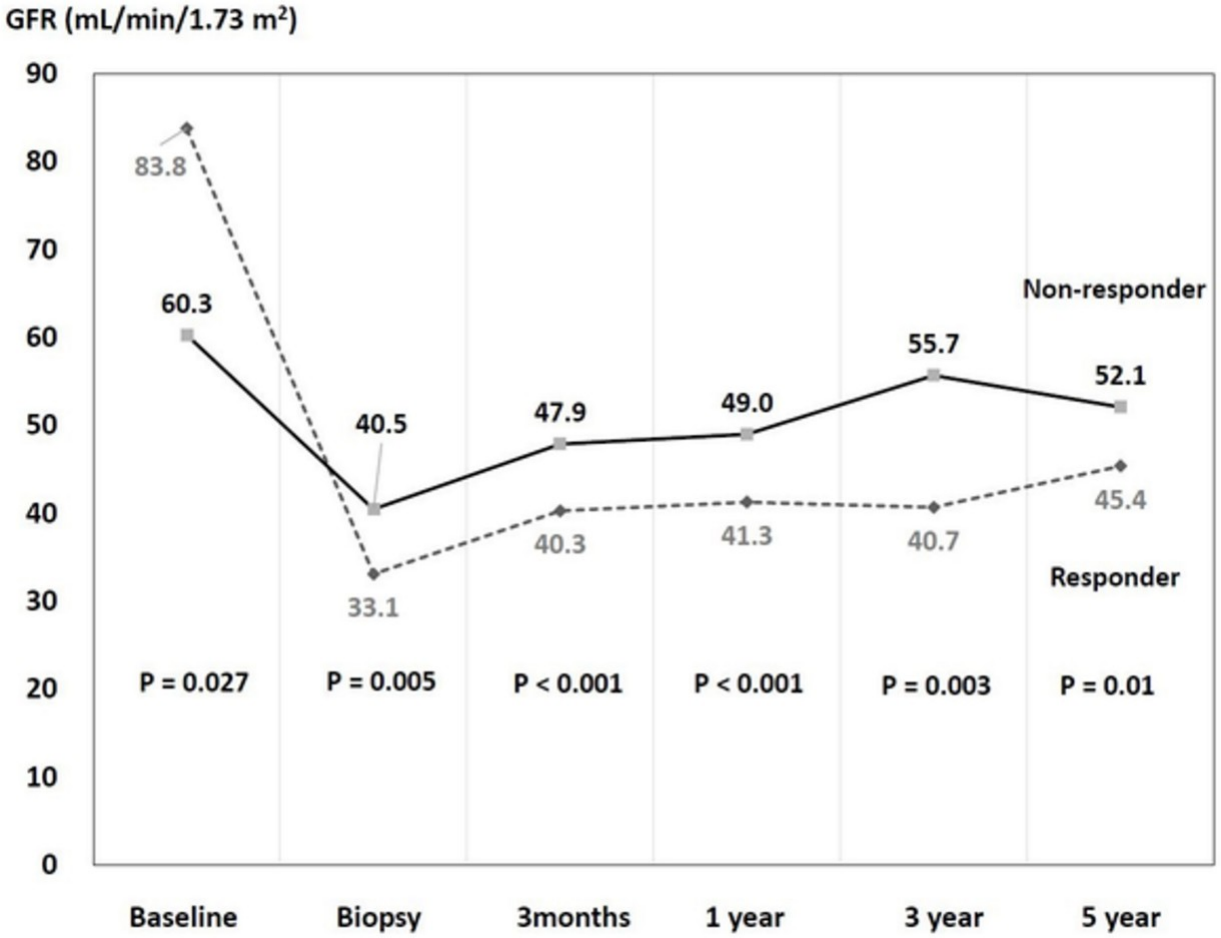
Changes in the estimated glomerular filtration rates.

The clinical characteristics of the patients at the time of TCMR are shown in **Table 2.** Rejection time since KT (median [IQR]) in non-responders and responders were 24 (16–42) and 11 (3–33) months, respectively (*P =* 0.008). Of the non-responders, 19 (86%) had grade I acute TCMR and 3 (14%) had grade II acute TCMR; of the responders, 34 (83%), 6 (15%), and 1 (2%) patients had grade I, II, and III acute TCMR, respectively. GFR at biopsy was significantly higher in the responders (40.5 [35.7–46.2]) than in the non-responders (33.1 [24.4–39.5]; *P =* 0.005); the changes in GFR from baseline to the time of biopsy was significantly greater in the non-responders (36.7 [22.6–47.2]) than in the responders (16.3 [12.7–21.9]; *P <* 0.001). After rejection treatment, the GFR values in the non-responders remained lower than those in the responders (**Fig 1**).

**Table 2:** Clinical characteristics at the time of acute T cell-mediated rejection.

	Non-responder (N = 22, 34.9%)	Responder* (N = 41, 65.1%)	*P*-value
Rejection time since transplantation (months)	24 (16–42)	11 (3–33)	0.008
TCMR Banff grade			0.013
IA	4 (18.2)	20 (48.8)	
IB	15 (68.2)	14 (34.1)	
IIA	1 (4.5)	6 (14.6)	
IIB	2 (9.1)	0 (0.0)	
III	0 (0.0)	1 (2.4)	
DSA positive	4 (18.2)	4 (9.8)	0.34
Maximal DSA MFI	4796 (4592–5000)	1363 (1061–4434)	0.25
GFR at biopsy (mL/min/1.73 m^2^)	33.1 (24.4–39.5)	40.5 (35.7–46.2)	0.005
ΔGFR^$^ (mL/min/1.73 m^2^)	36.7 (22.6–47.2)	16.3 (12.7–21.9)	< 0.001
Rejection treatment			
Steroid treatment	21 (95.5)	40 (97.6)	0.65
Steroid dose	2.1 ± 0.6	1.8 ± 0.6	0.10
Thymoglobulin	1 (4.5)	1 (2.4)	0.65
Thymoglobulin dose (mg)/ body weight (kg)	5.9	9.5	0.99
Recurrent rejection after treatment	14 (63.6)	12 (29.3)	0.008
TCMR only	9 (40.9)	8 (19.5)	
ABMR ± TCMR	5 (22.7)	4 (9.8)	
Recurrent rejection time since treatment (months)	27.5 (9.5–41.8)	49.0 (30.0–90.0)	0.012

Continuous data are presented as mean ± standard deviation, categorical data are presented as number (%), and non-normally distributed data are presented as median (interquartile range).

*Responders: patients whose serum glomerular filtration rate decreased by less than 30% from baseline value at three months after rejection treatment.

^$^ ΔGFR: baseline GFR–GFR at biopsy.

Abbreviation: TCMR, T-cell mediated rejection; DSA, donor-specific antibody; MFI, mean fluorescence intensity; GFR, *glomerular filtration rate; ABMR*, antibody-mediated rejection.

**Fig 2:**
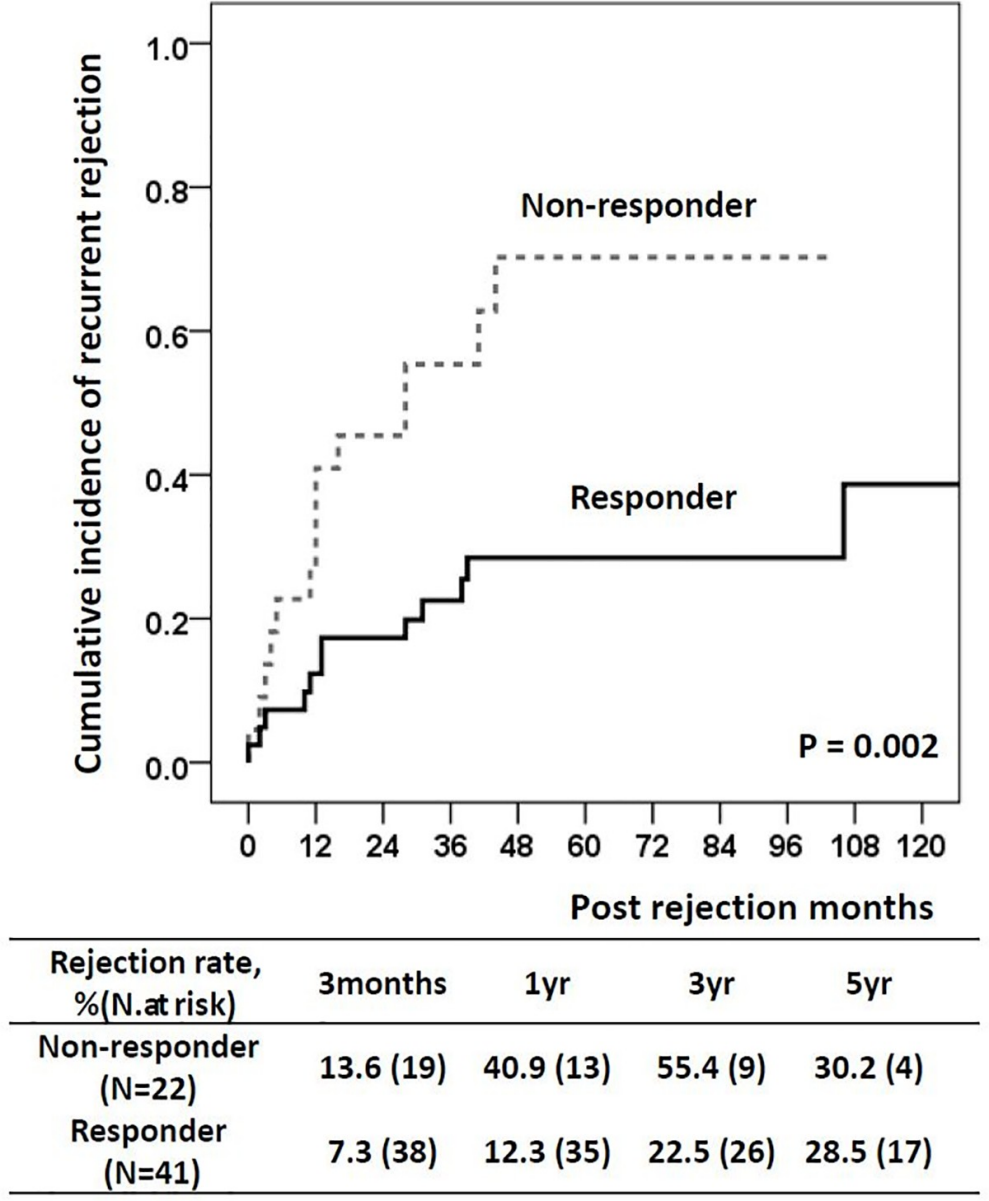
Cumulative incidence of recurrent rejection after rejection treatment.

The non-responders and responders did not show significant differences in the proportion of patients with positive DSA, maximal DSA median fluorescence intensity, steroid dose for pulse treatment, and the number of patients treated with ATG. The proportion of patients who showed any recurrent rejection following the first episode of acute TCMR was significantly higher in the non-responder group (64%) than in the responder group (29%; *P =* 0.008). The rejection time since treatment also shorter in the non-responders (27.5 months, IQR 9.5–41.8) than in the responders (49.0 months, IQR 30.0–90.0; *P =* 0.012). The cumulative incidence of recurrent rejection after the treatment also significantly higher in the non-responders (*P =* 0.002) (**Fig 2**).

The pathologic characteristics of the patients at the time of acute TCMR are shown in **Table 3**. The histological results showed that the proportion of patients with a tubulitis (t) score of 3 was significantly higher in the non-responder group (86%) than in the responder group (46%; *P =* 0.008). The interstitial fibrosis/tubular atrophy (IF/TA) score was generally higher in the non-responders than in the responder group (*P =* 0.013); whereas 23% and 46% of the non-responder group had scores of 1 and 2, respectively, 59% of the responder group had a score of 1 and only 12% had a score of 2. Otherwise, the two groups did not show significant differences in the Banff scores.

**Table 3:** Pathologic characteristics at the time of acute T cell-mediated rejection.

	Non-responder (N = 22, 34.9%)	Responder* (N = 41, 65.1%)	*P*-value
Interstitial inflammation (i) score			0.76
0	0 (0)	0 (0)	
1	0 (0)	1 (2.4)	
2	9 (40.9)	17 (41.5)	
3	13 (59.1)	23 (56.1)	
Tubulitis (t) score			0.008
0	0 (0)	0 (0)	
1	0 (0)	1 (2.4)	
2	3 (13.6)	21 (51.3)	
3	19 (86.4)	19 (46.3)	
Intimal arteritis (v) score			0.13
0	19 (86.4)	34 (82.9)	
1	1 (4.5)	6 (14.6)	
2	2 (9.1)	0 (0.0)	
3	0 (0.0)	1 (2.4)	
Glomerulitis (g) score			0.59
0	16 (72.7)	34 (82.9)	
1	5 (22.7)	5 (12.2)	
2	1 (4.5)	1 (2.4)	
3	0 (0.0)	1 (2.4)	
Peritubular capillaritis (ptc) score			0.66
0	11 (50.0)	27 (65.9)	
1	2 (9.1)	3 (7.3)	
2	7 (31.8)	9 (22.0)	
3	2 (9.1)	2 (4.9)	
Chronic allograft glomerulopathy (cg) score			0.29
0	22 (100.0)	39 (95.1)	
1	0 (0.0)	2 (4.9)	
2	0 (0.0)	0 (0.0)	
3	0 (0.0)	0 (0.0)	
Interstitial fibrosis/tubular atrophy (IF/TA) score			0.013
0	6 (27.3)	11 (26.9)	
1	5 (22.7)	24 (58.5)	
2	10 (45.5)	5 (12.2)	
3	1 (4.5)	1 (2.4)	
Vascular fibrous initial thickening (cv) score			0.76
0	8 (36.4)	15 (36.6)	
1	12 (54.5)	20 (48.7)	
2	2 (9.1)	4 (9.8)	
3	0 (0.0)	2 (4.9)	
Arteriolar hyalinosis (ah) score			0.44
0	7 (31.9)	19 (46.3)	
1	9 (40.9)	8 (19.5)	
2	5 (22.7)	13 (31.8)	
3	1 (4.5)	1 (2.4)	
C4d deposition in peritubular capillaries score			0.93
0	20 (90.9)	37 (90.2)	
1	2 (9.1)	4 (9.8)	
2	0 (0.0)	0 (0.0)	

Values are number (%).

*Responders: patients whose serum glomerular filtration rate decreased by less than 30% from baseline value at three months after rejection treatment.

### Risk factors associated with non-response and graft failure

**Fig 3:**
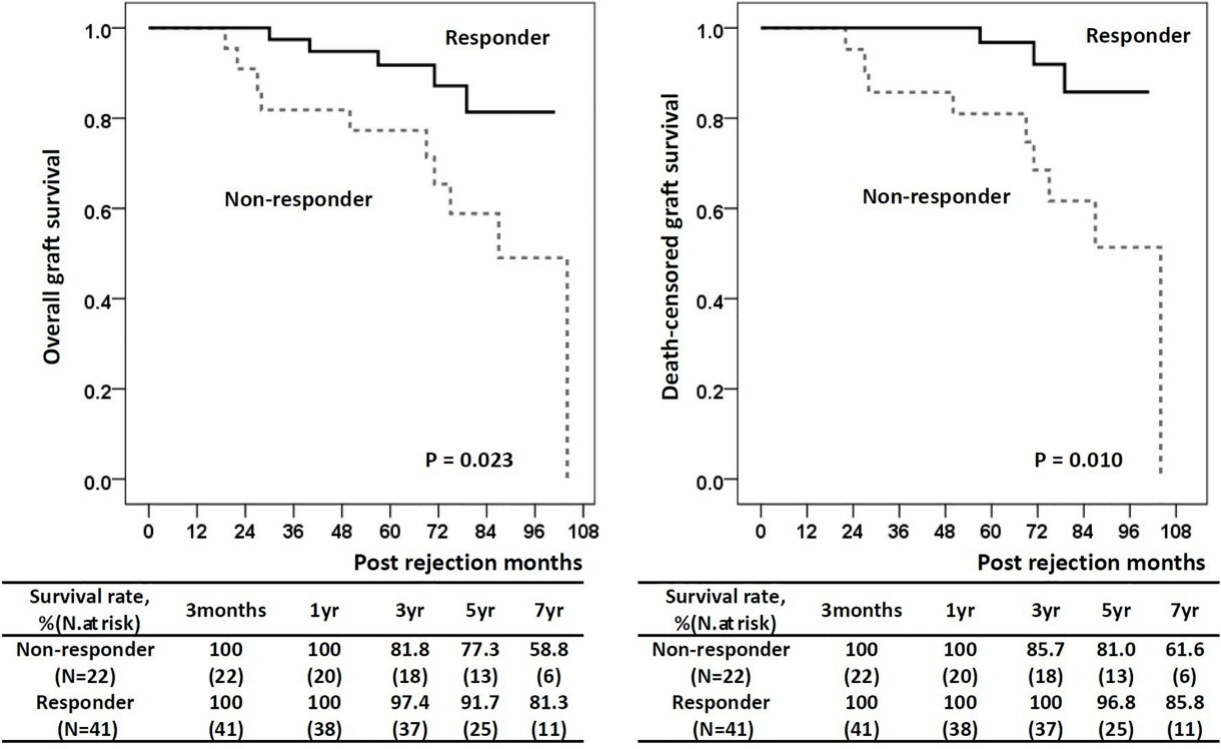
Overall and death-censored graft survival rate after rejection treatment.

Variables that showed a *P*-value of <0.1 on univariate analysis were included in multivariate analysis. The following variables were found to be significantly associated with non-response to rejection treatment: GFR at biopsy per 10 mL/min/1.73m^2^ (odds ratio [OR] = 0.33; 95% confidence interval [CI], 0.12–0.87; *P* = 0.025), ΔGFR (baseline GFR–GFR at biopsy) per 10 mL/min/1.73m^2^ (OR = 2.39; 95% CI = 1.25–4.56; *P =* 0.008), TCMR within 1 year vs. after 1 year (OR = 0.05; 95% CI = 0.01–0.52; *P =* 0.006), t score (OR = 50.72; CI = 3.07–839.02; *P =* 0.006), and IF/TA score (OR = 1.67; CI = 1.06–20.53; *P =* 0.041) (**Table 4**). We also evaluated the risk factors for graft failure (**Table 5**), and found that non-responder vs. responder (hazard ratio [HR] = 3.31; 95% CI = 1.08–10.09; *P =* 0.036) and lower GFR at biopsy per 10 mL/min/1.73m^2^ (HR = 0.56; 95% CI = 0.34–0.93; *P =* 0.026) were independent risk factors in the multivariate analysis. The responder group showed a significantly superior overall GS rate compared with the non-responder group (*P =* 0.023) (**Fig 3**).

**Table 4:** Risk factors associated with non-response to rejection treatment.

	Univariate analysis		Multivariate analysis	
	OR (95% CI)	*P* value	OR (95% CI)	*P*-value
Age	1.03 (0.98–1.08)	0.24	–	–
Female vs. male	1.10 (0.32–3.74)	0.88	–	–
ABO incompatibility	0.58 (0.11–3.17)	0.53	–	–
HLA-A, B, DR mismatch	0.91 (0.67–1.24)	0.56	–	–
PRA class I	1.03 (0.94–1.13)	0.49	–	–
PRA class II	1.09 (0.90–1.30)	0.38	–	–
Cyclosporin vs. Tacrolimus	1.53 (0.54–4.35)	0.42	–	–
GFR at biopsy per 10 mL/min/1.73m^2^	0.44 (0.24–0.82)	0.010	0.33 (0.12–0.87)	0.025
ΔGFR^$^ per 10 mL/min/1.73m^2^	1.92 (1.31–2.84)	0.001	2.39 (1.25–4.56)	0.008
TCMR within 1 year vs. after 1 year	0.17 (0.05–0.61)	0.006	0.05 (0.01–0.52)	0.005
TCMR after 2 year vs. within 2 year	0.56 (0.19–1.62)	0.28	–	–
TCMR IB vs. IA	0.38 (0.12–1.21)	0.11		
Interstitial inflammation (i) score	1.22 (0.45–3.29)	0.70	–	–
Tubulitis (t) score	7.20 (1.84–27.54)	0.004	50.72 (3.07–839.02)	0.006
Interstitial fibrosis/tubular atrophy (IF/TA) score	1.82 (0.92–3.56)	0.085	4.67 (1.06–20.53)	0.041

*Responders: patients whose serum glomerular filtration rate decreased by less than 30% from baseline value at three months after rejection treatment.

^$^ ΔGFR: baseline GFR–GFR at biopsy.

Abbreviation: PRA, panel reactive antibody; GFR, *glomerular filtration rate;* TCMR, T-cell mediated rejection.

**Table 5:** Risk factors associated with graft failure.

	Univariate analysis		Multivariate analysis	
	HR (95% CI)	*P*-value	HR (95% CI)	*P*-value
Non-responder vs. responder*	4.45 (1.51–13.12)	0.007	3.31 (1.08–10.09)	0.036
GFR at biopsy per 10 mL/min/1.73m^2^	0.49 (0.30–0.79)	0.003	0.56 (0.34–0.93)	0.026
ΔGFR^$^ per 10 mL/min/1.73m^2^	1.16 (0.89–1.49)	0.27	–	–
TCMR within 1 year vs. after 1 year	0.48 (0.17–1.40)	0.18	–	–
TCMR after 2 year vs. within 2 year	0.59 (0.19–1.80)	0.35	–	–
Interstitial inflammation (i) score	0.85 (0.31–2.32)	0.76	–	–
Tubulitis (t) score	1.95 (0.63–6.01)	0.25	–	–
Interstitial fibrosis/tubular atrophy (IF/TA) score	0.93 (0.48–1.82)	0.84	–	–

*Responders: patients whose serum glomerular filtration rate decreased by less than 30% from baseline value at three months after rejection treatment.

^$^ ΔGFR: baseline GFR–GFR at biopsy.

Abbreviation: PRA, panel reactive antibody; GFR, *glomerular filtration rate;* TCMR, T-cell mediated rejection.

### Patient demographics and MMDx results in molecular diagnosis group

A total of 23 patients underwent molecular diagnosis and were divided into three groups according to the histological results as follows: 7 (30%) patients with no rejection, 4 (17%) patients with pure acute TCMR, and 12 (52%) patients showing ABMR with or without TCMR (**Table 6**). The 7 patients with no rejection in histology also showed no rejection in TCMR and ABMR MMDx scores, except for one patient with a TCMR score of 0.24. In contrast, all 4 patients with acute pure TCMR showed discordant results in the MMDx. All patients in the TCMR only group had TCMR MMDx scores of between 0 and 0.1. Two out of the four patients in the TCMR group had ABMR MMDx scores of 0.2–0.5. In the ABMR group, 5 (42%) patients had ABMR MMDx scores of 0.2–0.5 and 7 (58%) patients had ABMR MMDx scores of > 0.5.

**Table 6:** Baseline and clinical characteristics of the patients in the molecular diagnosis group.

	No rejection (N = 7, 30.4%)	TCMR only (N = 4, 17.4%)	ABMR^$^ (N = 12, 52.2%)	*P*-value
Mean age (years)	46.4 ± 16.1	47.5 ± 10.1	50.1 ± 11.0	0.82
Female sex	5 (71.4)	4 (100.0)	6 (50.0)	0.18
Body mass index (kg/m^2^)	25.0 ± 4.9	28.8 ± 5.9	23.9 ± 2.9	0.15
HLA-A, B, DR mismatch	2.7 ± 1.7	2.8 ± 1.9	2.9 ± 1.4	0.96
PRA class I	0 (0–0)	26.0 (5.8–31.3)	17.5 (0–80.5)	0.05
PRA class II	0 (0–0)	4.0 (0–38.0)	0 (0–72.0)	0.82
Rejection time since transplantation (months)	28.0 (15.0–59.0)	42.5 (18.3–72.0)	29.0 (13.3–53.0)	0.89
TCMR Banff grade				–
No	7 (100.0)	0 (0.0)	9 (75.0)	
IA	0 (0.0)	3 (75.0)	1 (8.3)	
IB	0 (0.0)	1 (25.0)	2 (16.7)	
TCMR MMDx score^a^				–
0–0.1	6 (85.7)	4 (100.0)	4 (33.3)	
0.1–0.3	1 (13.3)	0 (0.0)	3 (25.0)	
0.3–0.6	0 (0.0)	0 (0.0)	2 (16.7)	
> 0.6	0 (0.0)	0 (0.0)	3 (25.0)	
ABMR MMDx score^b^				–
0–0.2	7 (100.0)	2 (50.0)	0 (0.0)	
0.2–0.5	0 (0.0)	2 (50.0)	5 (41.7)	
> 0.5	0 (0.0)	0 (0.0)	7 (58.3)	

Continuous data are presented as mean ± standard deviation, categorical data are presented as number (%), and non-normally distributed data are presented as median (interquartile range).

^$^ ABMR: Antibody-mediated rejection with/without TCMR.

^a^ TCMR MMDx score was classified as normal, mild, moderate, and severe using the cut off of 0.1,0.3, and 0.6, respectively.

^b^ ABMR MMDx score was classified as normal, mild, and severe using the cut off of 0.2 and 0.5, respectively.

Abbreviation: PRA, panel reactive antibody; TCMR, T-cell mediated rejection; ABMR, Antibody-mediated rejection; MMDx, molecular microscope diagnostic system.

**Fig 4:**
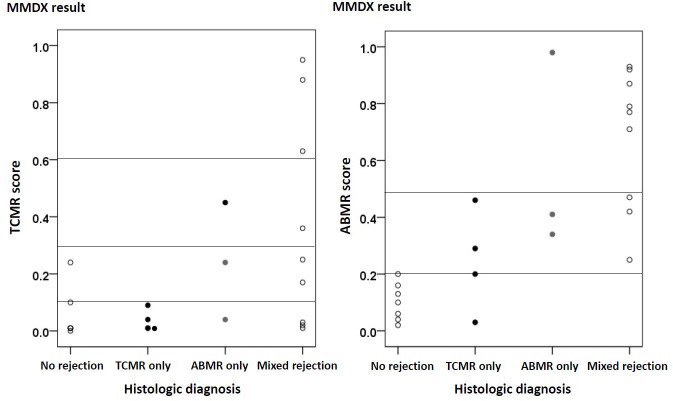
Association between the molecular microscope diagnostic system and the histological diagnosis.

The association between histologic diagnosis and the molecular diagnosis indicated that the ABMR MMDx score was consistent with the histologic ABMR (**Fig 4**). Molecular assessment using MMDx showed a good correlation with histologic diagnosis in ABMR (accuracy = 91.3% / positive predictive value [PPV] = 85.7% / negative predictive value [NPV] = 100%), but not in TCMR (accuracy = 47.8% / PPV = 22.2% / NPV = 64.3%) (**Table 7**).

**Table 7:** Agreement of histologic diagnosis with molecular diagnosis.

	TCMR	No TCMR	ABMR	No ABMR
TCMR MMDx score ≥ 0.1	2	7		
TCMR MMDx score < 0.1	5	9		
ABMR MMDx score ≥ 0.2			12	2
ABMR MMDx score < 0.2			0	9
TCMR MMDx score ≥ 0.1	Accuracy = 47.8% / PPV = 22.2% / NPV = 64.3%			
ABMR MMDx score ≥ 0.2	Accuracy = 91.3% / PPV = 85.7% / NPV = 100%			

Abbreviation: TCMR, T-cell mediated rejection; ABMR, antibody-mediated rejection; MMDx, molecular microscope diagnostic system; PPV, positive predictive value; NPV, negative predictive value.

## Discussion

In this study involving 63 patients with pure acute TCMR, we found that solitary TCMR without proof of ABMR was a significant risk factor for graft failure in patients who did not respond to rejection treatment. Acute TCMRs that developed within one year after KT tended to respond well to steroid treatment, while those developing after one year following KT did not. By studying an additional 23 patients with microarray analysis, we found that the histologic diagnosis of acute ABMR was closely correlated with the molecular diagnosis. However, all 4 cases of acute pure TCMR in histologic evaluation did not show any evidence of TCMR in MMDx. Therefore, clinicians treating patients who develop acute pure TCMR at one year after transplant in the pathological examination should consider the possibility of hidden ABMR or other causes of azotemia, especially when the patients are not responding well to steroid treatment.

As TCMR remains a significant clinical subject in the aspect of a bidirectional relationship with ABMR, the concept of chronic active TCMR was introduced [7–9]. One report showed that concurrent acute TCMR in recipients with biopsy-proven ABMR was an independent risk factor for graft loss [9]. Another study suggested that persistent TCMR induced graft interstitial inflammation and fibrosis, thus leading to allograft failure [7]. Additionally, recent studies have demonstrated a link between pure TCMR, the development of de novo DSAs, and late graft failure due to chronic active ABMR [18, 19]. In our study, four out of nine patients who experienced AMBR after rejection treatment developed de novo DSAs. Two patients recovered graft function after rejection treatment. In the other two patients, however, repeated AMBR resulted in graft failure due to chronic active ABMR. The 2017 Banff classification introduced the chronic active TCMR, which was defined as inflammation in the area of interstitial fibrosis and tubular atrophy with moderate or severe tubulitis [8]. In a study that reviewed 963 indication biopsy samples, chronic histological TCMR lesion within one year after KT was revealed as an independent risk factor of long-term GS [20]. Recently, Bouatou et al. reported the impact of pure acute TCMR according to the treatment response based on composite prognostic factors such as GFR, inflammation in the IF/TA area, and anti-HLA DSAs [10]. Although the definition of treatment response was not entirely consistent with that used in our current study, both studies reported similar results in terms of the risk factors of non-response, including time since KT and IF/TA. The advantage of our study is that the median time (39.0 months) from KT to rejection was longer than that in the above-mentioned study (39.0 vs. 3.5 months, respectively), which enabled us to analyze the clinical significance of acute pure TCMRs that are reported more than one year after transplant. Among 27 patients who occurred TCMR within one year after transplantation, only 4 (18%) did not respond to mPD pulse treatment. However, acute TCMR developing after one year following KT was an independent risk factor of non-response to rejection treatment.

Molecular assessment using a microarray of biopsies in KT is a feasible and accurate method for complementing histologic diagnosis [16]. Discrepancies between molecular and histological diagnosis in TCMR mainly develop in inflammatory conditions due to acute kidney injury, ambiguous isolated v-lesions, scarred tissues, and combined polyomavirus related lesions [13, 21]. The recent study also demonstrated that MMDx scores had a good correlation with histology. However, TCMR in histologic diagnosis increased discrepancies [22]. ABMR may induce tubulointerstitial inflammation and thus mimic TCMR—however, this may be missed by the site-specific nature of histologic diagnosis [23, 24]. In three (n = 1 in the responder group and n = 2 in the non-responder group) cases of our study who showed recurrence of ABMR within three months after initial TCMR, there was a possibility that the cause of TCMR was related to ABMR. The disagreement predominantly occurred in the biopsy samples that had been obtained either immediately post-operative or beyond one year post-operative [24]. Halloran group reported that while pure TCMR became rare overtime and disappeared by 10 years after transplant, ABMR continued to appear even decades after KT [2]. In our study, the four patients in the molecular diagnosis group who were diagnosed with pure TCMR had a median rejection time of 42.5 months and showed a low correlation with MMDx results. Considering that the results of the ABMR were highly consistent between histologic and molecular diagnosis, this disagreement in TCMR is not likely a result of an error during histological analysis. Among these four cases, one with Banff scores of interstitial inflammation (i) 2 and t2 recovered from azotemia after steroid treatment; one case with Banff scores of i2 and t3 was expected to be related to a myocardial infarction that occurred a week after a biopsy. Another patient with TCMR IA had BK viremia. However, the result of BK virus immunostaining (Simian virus 40) was negative. Finally, one patient had a severe chronic change which included features of acute TCMR with Banff scores of i2 and t2; that hindered the diagnosis of acute rejection. Since all four patients showed rapid recovery of GFR after treatment, there was a possibility that non-specific inflammation had been diagnosed as TCMR. Using treatment response as an indicator of accurate diagnosis should be done with caution because clinicians try various ways to improve clinical outcomes such as immunosuppressant adjustment, hydration, and correction of other possible factors that can suppress the kidney function. In addition, T-cell reaction and B-cell reaction are not separate events, and steroid treatment can affect the kidney function through multiple mechanisms [25, 26].

Our study showed that GFR decrement at the time of biopsy, t score, IF/TA score, and TCMR occurred one year after transplant were independent risk factors of non-response to rejection treatment. Late TCMR has been suggested as a predictor of low GS in previous studies [27, 28]. Recently, Mayrdorfer et al. suggested that TCMR might be a contributing factor in about one-third of early and late graft failures [29]. For over a long term after transplantation, nonimmunologic factors, including chronic toxicity, had great importance in graft function [30]. In addition, chronic histological damage markedly affects graft survival [20]. Therefore, acute TCMR developing on the graft with chronic damage may be relatively difficult to recover from rejection. Similarly, the non-responder group in our study had a significantly higher IF/TA score than the responder group, and IF/TA was a significant risk factor associated with non-response. There are no definite criteria for distinguishing between early TCMR and late TCMR; in our study, TCMR developing at one year after KT was a significant risk factor for non-response. However, we did not find the statistical significance of risk for non-response to treatment when the cut value was divided two years after the transplant. The Banff t lesion showed a 50.7 fold increase for the risk of non-response in our study, but the i score showed no significance in multivariate analysis. Nankivell et al. showed that interstitial inflammation by mononuclear cells is the initial event of cellular rejection that results in adverse histological and functional outcomes, and is thus a key diagnostic feature of TCMR. However, the t-score replaced i score as a significant predictor of progressive fibrosis [21]. Although Sellares et al. suggested that both i and t scores had no associations with long-term GS, another study showed that intimal arteritis (v) and t score were independent indicators of GS, which is similar to our study [27, 28]. The low GFR value at biopsy and large decrement of GFR from baseline were also independent predictors of non-response to rejection treatment.

We used GFR, a simply measurable value, as a single indicator for dividing the responders from the non-responders, and the cut-off value of 30% decrement from the baseline after rejection treatment was chosen as it was the point after which significant differences in GS were noted, and the recovery period of three months was determined in a previous study of Bouatou et al. [10] We do not consider this value as an absolute cut-off point that can be generally applied. Our objective was to demonstrate the tendency, among patients with less GFR recovery after rejection treatment, for poor long-term graft survival, and to suggest a potentially significant cut-off value. GFR values 3 months after treatment could be affected by other relevant causes. There were two patients with urinary tract infection and diarrhea, which may have affected the GFR. In these two patients, the differences between the GFR measured at 3 months vs. the next value were 10 and 8 mL/min/1.73 m^2^, respectively. However, it was difficult to prove that the GFR changes were caused by these conditions. If we used GFR values determined after recovery from these conditions, this would have reflected selection bias. Additionally, we did not find GFR values that significantly deviated from the GFR trend. Therefore, we chose the most closely measured GFR values at 3 months after treatment without exception. After rejection treatment, the leading causes of graft failure were recurrent rejection and kidney function deterioration without recovery except for three patients, in whom graft failure occurred due to subarachnoid hemorrhage, pneumonia, or lung cancer. CD4 T cells activation can act as effector cells for initiating humoral responses [31]. Among the 26 patients with recurrent rejection after treatment, five (23%) in the non-responder group and four (10%) in the responder group developed ABMR. Uncontrolled acute pure TCMR seemed to induce recurrent rejection including ABMR. Among eight DSA-positive patients, seven were C4d-negative without other evidence of antibody interactions with vascular endothelium. One patient who had DSAs with grade 1 C4d positivity did not demonstrate histologic evidence of acute tissue injury. Given that only one of these eight patients developed ABMR after steroid treatment, it is believed that the ABMR that occurred after treatment might not simply have been due to mistaken interpretation of histologic findings.

Our study has several limitations. First, the number of patients included in this study was not large enough to obtain enough statistical power. However, considering the low incidence of acute pure TCMR, especially after a long time following transplant, the number of patients used in this study is quite large compared with those used in other recent studies Second, to properly analyze non-responses to treatment, renal biopsy samples should be taken after patients complete steroid treatment. There might be differences between histologic responses and clinical outcomes. Our study suggested clinical findings that affect long-term GS after treatment of pure TCMR. Third, we did not perform the histological diagnosis of i and t scores in IF/TA lesions because biopsy reviews were performed prior to 2017 [8]. Our study focused on acute pure TCMR lesion rather than chronic changes. Fourth, considering that this study was a single-center study that solely included Asian patients, caution is needed when generalizing our results to patients with different genetic and medical circumstances that affect clinical outcomes. Lastly, patients in the molecular diagnosis group were not those included in the histologic analysis group, which led to difficulties in interpreting the results. Nevertheless, we presume that enough information about the possibility of misdiagnosis in acute pure TCMR at long-term after transplant was obtained through molecular diagnosis.

## Conclusions

In conclusion, we report that pure acute TCMRs that do not respond to treatment was a significant risk factor of graft failure. Molecular analysis using MMDx showed that acute pure TCMR at one year after transplant in the histologic report should be interpreted cautiously. Our results suggest that considering TCMR as a significant factor may be helpful in improving long-term GS in the era of ABMR. There is a need for planned strategies for monitoring and maintenance methods after rejection treatment.

## Supporting information

S1 Checklist (DOCX)

S1 File Raw data of this study (SPSS). (SAV)

S2 File Raw data of this study: MMDx subgroup (SPSS). (SAV)
